# Infarct delineation in patients with acute myocardial infarction using the tractographic propagation angle and late gadolinium enhancement

**DOI:** 10.1186/1532-429X-17-S1-P16

**Published:** 2015-02-03

**Authors:** Choukri Mekkaoui, Marcel P Jackowski, Christian T Stoeck, William J Kostis, Fabricio Pereira, Sebastian Kozerke, David E Sosnovik

**Affiliations:** 1 Harvard Medical School - Massachussets General Hospital, Charlestown, MA, USA; 2 University of São Paulo, São Paulo, Brazil; 3 ETH Zurich, Zurich, Switzerland; 4 CHU Nimes, Nimes, France

## Background

Currently used techniques to quantify late gadolinium enhancement (LGE) include an intensity threshold 5 standard deviations (5-SD) above normal myocardium and the full width at half maximum (FWHM) of the histogram [1,2]. Validation of these segmentation schemes can be performed *ex vivo* using tetrazolium tetrachloride (TTC). However, no analogous gold standard metric exists *in vivo*. We recently introduced the tractographic propagation angle (PA) [3], and showed that a PA threshold of 4° in infarcted hearts produces a distribution that corresponds very closely to that of TTC staining [4]. Here, we use PA maps in patients with myocardial infarction (MI) to evaluate the standard FWHM and 5-SD metrics. In addition, we introduce a new metric based on a Gaussian fit of the LGE image histogram.

## Methods

Patients with acute MI (n=3) were imaged at the diastolic sweet spot on a 1.5 T scanner with a diffusion-encoded stimulated echo EPI sequence using the following parameters: resolution 2x2x8 mm^3^, b-value of 500 s/mm^2^, 10 diffusion-encoding directions, and 8 averages. LGE was performed using a 2D inversion recovery gradient echo sequence. Mean diffusivity (MD), fractional anisotropy (FA), helix angle (HA), and PA were calculated from the dyadic diffusion tensor. LGE of 16 short-axis slices were available for analysis. In addition to computing the 5-SD and FWHM, a Gaussian fit of each image histogram was performed and a threshold applied in 10% increments (G10-G100) of the total area.

## Results

**Figure 1:**
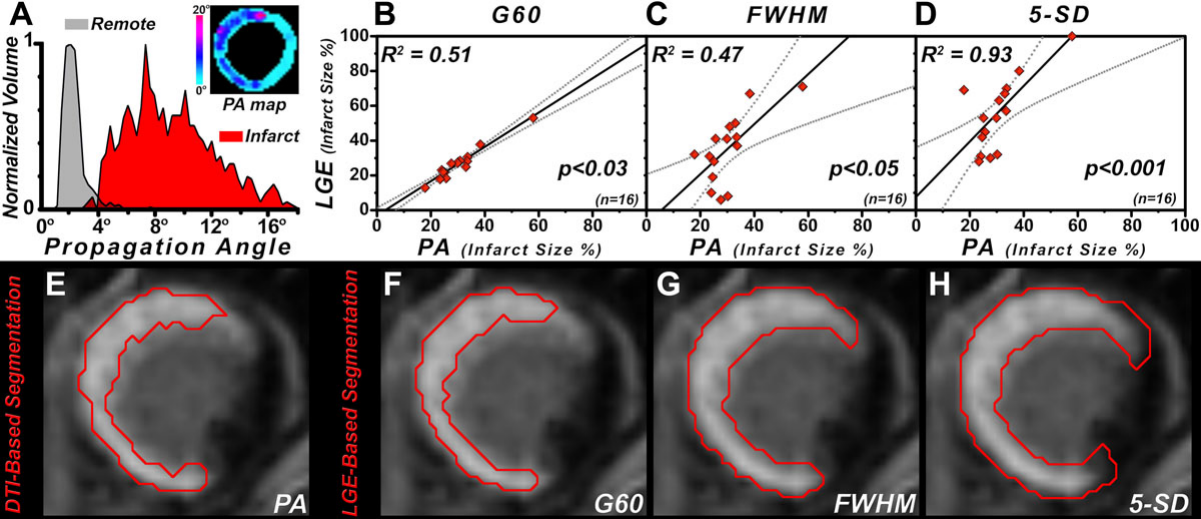
(A) Histogram of propagation angle (PA) values with discrimination of infarcted (PA > 4°) and remote (PA ≤ 4°) myocardium. Inset shows a PA map of a left ventricular short-axis slice illustrating the increase in PA in the infarct. (B-D) Least-squares fit of infarct size by G60, FWHM, and 5-SD *versus* that of PA. (E-H) Segmentation of infarcted myocardium by PA, G60, FWHM, and 5-SD. The 5-SD and FWHM metrics include a portion of the border zone and therefore overestimate the extent of the infarct.

**Figure 2:**
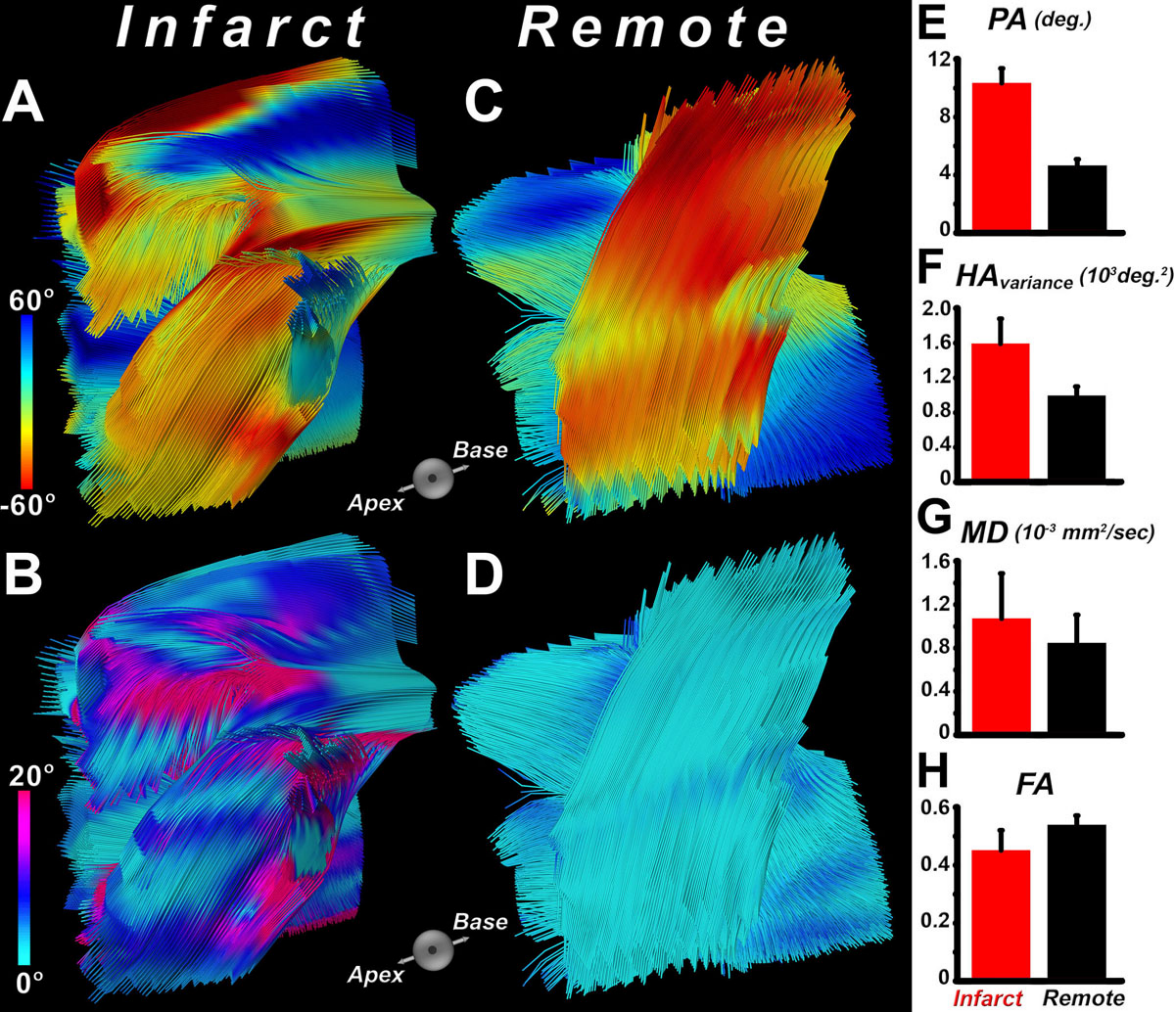
Tractograms of the infarcted region color-coded by (A) the helix angle (HA), and (B) propagation angle (PA). Tractograms of the remote zone color-coded by (C) the HA, and (D) PA. While myofibers in the remote zone are coherent and follow the typical transmural helical orientation, myofibers in the infarct zone exhibit disarray leading to an increase in PA. Bar plots comparing (E) PA, (F) HA_variance_, (G) MD, and (H) FA reveal significant differences in microstructure and myofiber organization between the infarcted and remote zones.

In normal myocardium PA is typically less than 4° (Figure 1A). Of all LGE-based metrics, G60 demonstrated the closest relationship to PA (Figures 1B-D). Figures 1E-H depict infarct segmentation by PA, G60, FWHM, and 5-SD; the latter two overestimating infarct size. Microstructural characterization of the infarct and remote zones was performed using DTI-tractography, color-coded by HA and PA (infarct zone: Figures 2A-B; remote zone: Figures 2C-D). PA, HA_variance_, and MD were higher in the infarct compared to the remote zone, while FA was lower (Figures 2E-H).

## Conclusions

A Gaussian fit using 60% of the LGE histogram area (G60) improves infarct segmentation and correlates well with a PA threshold of 4°. PA provides valuable information for infarct detection and microstructural characterization of the myocardium, can be assessed *in vivo* without exogenous contrast, and could prove valuable in a broad range of cardiovascular diseases.

## Funding

N/A.
